# Biomimetic Gut Model Systems for Development of Targeted Microbial Solutions for Enhancing Warfighter Health and Performance

**DOI:** 10.1128/mSystems.00487-20

**Published:** 2020-10-27

**Authors:** Lauren M. Brinkac, Nandita Rahman, Loun-Loun Chua, Sterling Thomas

**Affiliations:** a Noblis, Reston, Virginia, USA; Harvard Medical School

**Keywords:** coculture, gut microbiome, *in vitro*, gut model systems, warfighter, operational readiness, microbial-host interactions, enteric infection, diarrheal disease

## Abstract

The human gut microbiome plays a vital role in both health and disease states and as a mediator of cognitive and physical performance. Despite major advances in our understanding of the role of gut microbes in host physiology, mechanisms underlying human-microbiome dynamics have yet to be fully elucidated. This knowledge gap represents a major hurdle to the development of targeted gut microbiome solutions influencing human health and performance outcomes. The microbiome as it relates to warfighter health and performance is of interest to the Department of Defense (DoD) with the development of interventions impacting gut microbiome resiliency among its top research priorities. While technological advancements are enabling the development of experimental model systems that facilitate mechanistic insights underpinning human health, disease, and performance, translatability to human outcomes is still questionable. This review discusses some of the drivers influencing the DoD’s interest in the warfighter gut microbiome and describes current *in vitro* gut model systems supporting direct microbial-host interactions.

## Abstract (precis)

The human gut microbiome plays a vital role in both health and disease states and as a mediator of cognitive and physical performance. Despite major advances in our understanding of the role of gut microbes in host physiology, mechanisms underlying human-microbiome dynamics have yet to be fully elucidated.

## INTRODUCTION

The human gut microbiome is comprised of over 100 trillion microorganisms (microbes or microbiota) inhabiting the gastrointestinal (GI) tract (1); these microbes influence human physiology, metabolism, nutrition, and immune function. Disruption to the gut microbiota, known as dysbiosis, is defined as (i) loss of beneficial bacteria, (ii) overgrowth of potentially pathogenic bacteria, and (iii) loss of overall bacterial diversity; in most cases, these types of dysbioses occur simultaneously, such as those caused by antibiotic treatments, physiological stress, or diet (2). Dysbiosis is linked with GI conditions such as inflammatory bowel disease (IBD) and obesity (3), as well as a strong correlation with brain-, anxiety-, and trauma-related disorders (4).

The intestinal barrier acts as a selective facilitator for surveillance and response to agents interacting within its mucosal sites, often contingent upon the landscape of the host’s microbiome (5). Considered one of the most heavily innervated mucosal surfaces (6) in the body, the GI tract is implicated in dynamic neuroimmune host defense, where gut microbiota play a role in the development and functional maturation of the gut immune response (7). Mucosal neurons serve as sensors for internal environmental changes and signals (6) in the gut. Many of these neuronal signals elicit or inhibit immunoregulatory responses that are implicated in complications (such as vomiting and diarrhea) that affect both mental and physiological readiness (8) as the host attempts to eliminate harmful agents from the body (5, 9). All of these factors highlight the impact of microbiota in the mucosal region of the gut in relation to host defense and how these interactions could potentially be an etiological factor in complications such as gastrointestinal maladies.

While there is no single standard gut microbiome profile, recent research is elucidating the dominating factors for significant diversity in the human gut microbiome (10). In a global review, diet or diet-related factors (e.g., hygiene, parasitic load, environmental exposure, and dietary lifestyle) were the predominant factors in seven separate cohorts. These factors were driven by the types of local foods available to each population. Additionally, a further study of 1,135 participants from a more homogeneous Dutch population also showed diet as a significant factor for changes in diversity of the gut microbiome (11), with the most significant shifts in diversity being caused by the total quantity of carbohydrates consumed, consumption of plant proteins, and frequency of fruit consumption. This suggests that the human gut microbiome can be highly geographically localized based on types and content of foods available in the region, and any variation in nearby regions or behavioral selection (i.e., vegetarianism) will alter the microbiota. Changes in the gut microbiome begin occurring within a few hours to 1 day of a significant alteration in diet (12).

Fluctuating diversity in the gut can be a result of the previously mentioned factors and other functional needs; however, certain regions of the gut are able to maintain a relative level of compositional stability (13). A contributing factor for these selective differences in gut microbiome diversity is the composition of the host epithelium (14). The small intestine (SI), consisting of a single layer of epithelial cells and a mucosal layer, represents a physical barrier against the environment (15). The SI epithelium includes cell types for absorption of nutrients arising both from the digestive process and from gut microbiome metabolites (16). Additionally, the goblet cells of the SI epithelium produce and maintain a mucin layer that protects the epithelium from the gut microbiome and provides a selective atmospheric oxygen environment for growth of specific bacterial classes (14, 17). This layer is critical for the interaction between the gut and the microbiome by allowing the transfer and absorption of metabolites and protecting the epithelial cells from bacterial invasion.

Given the intricate system of the human gut microbiome and its collection of interdependencies, the design of a more accurate model of the human gut and microbiome that includes physical interactions of the epithelial cells and mucosa with the microbiome is required to effectively focus on opportunities applicable to U.S. Department of Defense (DoD) warfighter concerns.

In the next section, we discuss important studies and insights obtained from research in warfighters, specifically the current and future opportunities to model the intricacies of the gut microbiome and its interdependencies for increased accuracy.

## ROLE OF GUT MICROBIOTA IN THE WARFIGHTER

**FIG 1:**
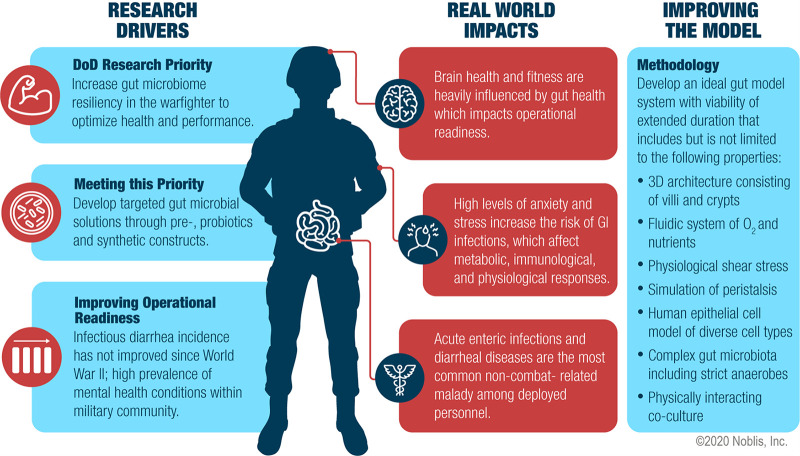
DoD gut microbiome research priorities and ideal *in vitro* model system characteristics.

In recent years, the DoD has shown particular interest in research focused on the influence of military-relevant stressors on interactions between the gut microbiome and warfighter biology, manipulation of the gut microbiome to influence warfighter health, and use of the gut microbiome as a biomarker of warfighter health status (18). One of the DoD’s top priority research outcomes for future studies includes building gut microbiome resiliency to pathogen infection to reduce the health burden of GI diseases and improve warfighter performance. Therefore, the continued development of microbiome interventions such as probiotics, prebiotics, or synthetic constructs that proactively influence the warfighter’s health, performance, or response to stress is of critical importance (Fig. 1).

### Enteric and diarrheal diseases.

Acute enteric infections and diarrheal diseases remain the most common non-combat-related malady among deployed personnel (19, 20). Recent estimates report a pooled incidence, consisting of both U.S. military and long-term travelers, of over 30 cases per 100 person-months (19). This incidence of acute diarrheal illness during initial deployment of warfighters has not decreased with the introduction of modern infectious disease treatments, as occurrence rates during Operations Iraqi Freedom and Enduring Freedom were remarkably similar to that experienced during World War II (WWII) in the same region (19, 21). Additionally, emerging evidence suggests that acute GI infections can develop into chronic ailments such as irritable bowel syndrome (IBS); those who endure high levels of anxiety and stress, such as military personnel, are at an increased risk (22). Thus, there is a critical need to advance mechanistic understanding of these illnesses to mitigate the substantial burden of afflictions in deployed military personnel, ultimately impacting operational readiness and effectiveness (18, 23).

The human gut microbiome is a critical component for protecting warfighters from acute diarrheal illness and potentially the progression to chronic disease. Among the bacterial causative agents of travelers’ diarrhea, enterotoxigenic and enteroaggregative Escherichia coli (ETEC and EAEC, respectively), Campylobacter jejuni, *Shigella*, and *Salmonella* species are the most common (24). The pathophysiology of diarrheal disease is multifactorial, but disruption of the intestinal epithelial barrier and dysregulation of mucosal immunity upon perturbation of the gut microbiome by enteric pathogens are a primary etiological factor (25).

Gut commensal microbiota are essential for maintaining human-microbiome homeostasis (26) and confer protection against diarrheal pathogen colonization through diverse mechanisms (27). Disruption of indigenous gut microbiota, such as through antibiotic use (28), may contribute to increased susceptibility to enteric infection (25). The extent to which an individual’s gut microbiota influences resistance to pathogen colonization remains to be fully elucidated, with limited studies having identified specific gut microbiota patterns associated with infection risk. In a human challenge study, *Sutterella* sp., Prevotella copri, and Bacteroides vulgatus were identified as gut microbiota having a potential protective effect against ETEC diarrheal disease in asymptomatic carriers, while individuals with gut microbiota enriched with Bacteroides dorei, *Prevotella* sp., Alistipes onderdonkii, *Bacteroides* sp. (Bacteroides ovatus), and *Blautia* sp. were associated with disease occurrence (29). *In vitro* gut model systems could help elucidate the mechanisms of colonization resistance by specific gut microbial taxa, aiding in the development of a prophylactic and treatment of travelers’ diarrhea, such as recombinant probiotics that neutralize heat-labile enterotoxin activity of ETEC (30). Therefore, in order to develop targeted microbial strategies that enhance defense against diarrheal infection, additional research is necessary to obtain a fully comprehensive understanding of the protective effect of specific microbial communities.

### Operational readiness and effectiveness.

Emerging research has granted a deeper understanding of the downstream effect that the gut microbiome has on physiological responses, particularly when faced with psychological, environmental, and physical stressors (18, 20). Warfighters are routinely subjected to these types of stressors in the field, where physiological and mental operational readiness is challenged (31, 32). Recent metagenomic and endurance studies of professional athletes versus sedentary subjects displayed marked increases in amino acid and antibiotic biosynthesis, as well as carbohydrate metabolism, and these changes were attributed to changes in gut microbiota (33), such as a higher prevalence of Veillonella atypica, which aids in lactate metabolism of marathon runners (34). Such physiological processes have a downstream effect on the rate of muscle turnover, potentially enhancing or diminishing fitness (31). Harnessing the dynamics between physiological stress and gut microbiota profiles could signal a promising entryway for performance enhancement (31) and, conversely, highlight the metabolic impacts of antibiotic use (33).

In instances such as endurance activities, physical and environmental stress responses have been linked to dysbiosis (35–37). Comorbidities such as heat stress and physical exertion are commonly encountered by warfighters and have been linked with increased GI permeability (38). Disruption in GI barrier integrity (38) can render the host vulnerable to, and more profoundly affected by, systemic endotoxemia caused by enteric bacteria (35, 39). Studies on military training in the Arctic demonstrate the relationship between physical stress and a shift in gut microbiota communities, marked by subsequent metabolite changes further enabling dysbiosis (37). Bioinformatics studies have quantitively illustrated how stress can reduce intestinal barrier integrity, altering gut microbiota composition, which in turn modulates immunoregulatory responses in the host (9, 40, 41). A compromised gut microbiota profile introduces disruption in the uptake of fluids, electrolytes, and vital nutrients, exerting negative impacts on exercise performance and recovery (42–44). Host-gut microbiota interactions are also implicated in the nervous system control of glucose, impacting another aspect of host metabolic efficiency (45). Pain is also associated with GI distress, which can introduce additional impairments in physical endurance (46). Through the brain-gut axis, gut microbiota can directly modulate afferent sensory neurons, eliciting pain (47, 48), including host nociceptor responses as seen in the case of Salmonella enterica serovar Typhimurium (6). There are implicit benefits to preventing dysbiosis, but a comprehensive understanding of these relationships could improve warfighter performance and also serve the general population, where the burden of GI diseases is also significant.

## EXISTING GUT MODEL SYSTEMS

Advancements in technology are promoting the development of experimental model systems that realistically mimic the microenvironment of the human gut, providing mechanistic insights underpinning human health, disease, and performance. While animal models are often used to investigate host-gut microbiota interactions and their contributions to host physiology and pathophysiology (49, 50), questions arise about their translatability to human outcomes (51, 52). Therefore, there is significant interest in developing *in vitro* models that closely resemble conditions of the human GI which can facilitate detailed mechanistic analysis in a tightly controlled, reproducible environment (53) (Table 1).

**TABLE 1:** Examples of current *in vitro* gut model systems supporting direct microbial-host interactions^*a*^

Properties	Gut model system				
	Apical anaerobic model	Enteroid- anaerobe coculture	Oxygen gradient device	Anaerobic intestine- on-a-chip	Anoxic-oxic interface- on-a-chip
Architecture	None	None	Crypts/microvilli	Villi	Villi
Mucus layer	No	Yes	Yes	Yes	Yes
Shear stress	Static	Static	Static	Static	Yes
Peristalsis	No	No	No	No	Yes
Epithelium integrity	TEER	TEER	TEER	Fluorescence	TEER
O_2_ gradient	Static	Active profusion	Passive diffusion	Active profusion	O_2_ gradient
Human epithelial model	Caco-2	HJEs	PCoESCs	Caco-2; HIMECs	Caco-2
Anaerobic microflora coculture	*F. prausnitzii*	B. thetaiotaomicron; *Blautia* sp.	*B. adolescentis*; *C. difficile*	B. fragilis	*B. adolescentis*; Eubacterium hallii
Complex microbial coculture	No	No	No	Yes	Yes
Direct interaction	Yes	Yes	Yes	Yes	Yes
Viability	8 h	24 h	24 h	5 days	7 days
Reference	56	58	63	64	65

a Abbreviations: TEER, transepithelial electrical resistance; Caco-2, human colon epithelial cell line; h, hours; HJEs, human jejunal enteroids; PCoESCs, primary human colon epithelial stem cells; HIMECs, human intestinal microvascular endothelial cells.

### *In vitro* coculture gut model systems.

An ideal *in vitro* human intestinal model can be described as containing all human-derived native gut epithelial cell types and all gut microbiota and recapitulating the three-dimensional (3D) complex tissue architecture, physiological shear, and cyclic stress forces acting on the epithelial cells (54). However, recapitulating a comprehensive spectrum of gut complexities and dynamics, including sustaining direct coculture of human tissue and complex microbial populations (aerobic and anaerobic), in a single *in vitro* model is challenging (54, 55). For example, the apical anaerobic model system uses a Transwell insert, consisting of a semipermeable membrane seeded with a monolayer of intestinal epithelial Caco-2 cells, which seals off a basal aerobic environment while also exposing its apical monolayer surface to an anaerobic environment of an external anaerobic chamber (56). This dual chamber design supports the aerobic requirement of Caco-2 cells while also facilitating their direct interaction with Faecalibacterium prausnitzii, an obligate anaerobe, residing in the apical compartment. However, due to its static oxygenated basal environment, coculture was limited to 8 h. Additionally, as Caco-2 cells did not originate from healthy epithelium (57), they may exhibit attributes different from normal tissue and subsequent host-gut microbiome dynamics may not accurately represent native intestine. A more recently developed enteroid-anaerobe coculture (EACC) system sustained coculture of anaerobes Bacteroides thetaiotaomicron and *Blautia* sp. with an established patient-derived intestinal enteroid cell line for up to 24 h. Coculture was extended in this Transwell-based system with the addition of a gas-permeable base used to oxygenate the basal aerobic compartment at defined physiological levels with an external tank (58). While human enteroids are a more physiologically relevant cell culture model, both the apical anaerobic model and EACC lack other physiologically relevant gut characteristics such as shear stress, simulation of peristalsis, and native architecture (i.e., villi, crypts, etc.), which reduces their extensibility.

Several different approaches have been taken to develop model systems that capture the native dynamics and architecture of the human gut environment. One technique employs biofabricated supports to recreate the structure of intestinal villi and crypts, which when seeded with epithelial cells present improved cellular physiology and differentiation (59, 60); others use a porous silk protein scaffolding system to construct a 3D tubular architecture representation of the intestines (61, 62). The oxygen gradient device mimics the topology of colonic crypts using collagen scaffolds for supporting the physical interaction of human primary colon epithelial stem cells and obligate anaerobes Bifidobacterium adolescentis and Clostridium difficile for up to 24 h through the generation of a self-sustaining, stable oxygen gradient across 3D crypt topology (63). The device employs an oxygen-impermeable plug creating an anaerobic environment by minimizing oxygen influx from above a monolayer of respiring epithelial cells separating apical anaerobic and basal aerobic compartments. Passive diffusion of atmospheric oxygen in direct contact with the basal compartment creates and maintains a physiological oxygen gradient within the system, eliminating the need for an external anaerobic environment or gas flow.

Recent microfluidic gut model systems include the anaerobic intestine-on-a-chip (64) and anoxic-oxic interface-on-a-chip (AOI) (65). These nonstatic devices support microbial cocultures that directly interact with host epithelial cells for extended periods (5 or 7 days, respectively) compared to those of other systems such as the Host-Microbiota Interaction (HMI) (66) and The Human Microbial Crosstalk (HuMiX) (67) models where direct interaction is currently not possible. Additionally, both the anaerobic intestine-on-a-chip and the AOI chip support the coculture of complex microbial populations and, in the case of the anaerobic intestine-on-a-chip, can support complex microbiota with primary human intestinal epithelium. Furthermore, the AOI device simulates the physiological flow and mechanical deformations representative of native gut epithelium which largely influence epithelial cell proliferation and differentiation and stability of gut microbiota (68). Transepithelial electrical resistance (TEER) was used in four of the five gut model systems described in Table 1; the fifth used a method based on fluorescence microscopy. The TEER methods tested the integrity of a monolayer of Caco-2 cells after growth over several days. The integrity of the cultured monolayer is critical for the survival of the epithelium after introduction of the bacterium, which led two studies to retest for monolayer integrity after introducing the bacterium. The valid readings were described as ranging between 500 Ω · cm^2^ and 4,000 Ω · cm^2^ (55). Finally, epithelial integrity and peristalsis have been shown to guide cell morphology (59, 60), and fluidic shear has been shown to increase differentiation in the epithelium over epithelial integrity alone.

## CONCLUSION AND FUTURE PERSPECTIVES

The gut microbiome is highly dynamic and correlated with multiple factors including diet, genetic makeup, stress, socioeconomic status, interactions between social and physical environments (42), and exercise (43). It fluctuates over time (13) and varies biogeographically across different sections of the gut (14). Diet exerts a large effect on the gut microbiota composition, but some bacteria thrive independently of dietary changes by colonizing the mucus layer as a reservoir and are maintained regardless of food intake (14). Thus, microbiome-host studies could target this mucus layer reservoir as a starting point to characterize gut resilience, and a systems biology approach to determine gut microbe interactions in enteric disease, pain, and the gut-brain axis must be considered (36).

While various models simulating the gut microbiome have been developed and studied, there still exists a need to create a reproducible simulated system that can accurately represent dynamic gas exchange, mechanical elements, and host-gut microbiome cross talk (69). These parameters are requisite in characterizing the links between physiological processes in the body and host gut microbiota. An *in vitro* model that simulates the physiological conditions of the GI tract, while sustaining the interaction between a coculture of intestinal epithelial cells and microbiota, would have the advantage of allowing detailed mechanistic analysis in a tightly controlled, reproducible environment.

The applications of an *in vitro* human intestinal model are numerous, but a direct application of interest is aiding in improving warfighter operational readiness and physiological performance. Enteric disease and its physiological and mental health implications remain a prominent etiological contributor in reducing warfighter performance, and those mechanisms have yet to be fully elucidated (19). Nutritional resource competition and gut dysbiosis can incite host defense mechanisms, where gut microbiota can serve to prevent exogenous pathogens from infecting the host or provoke them to infect the hose (7). These types of relationships are echoed in other physiological contexts that the warfighter can experience, such as interaction with the hypothalamic-pituitary-adrenal (HPA) axis and its implications for mental health, specifically posttraumatic stress disorder (PTSD) (70).

Creating comprehensive and reproducible *in vitro* human intestinal models would serve as a platform for researchers to rapidly illustrate and characterize these relationships and other physiological paradigms related to the gut microbiome. These can aid in development of therapeutic interventions or diagnostics, where bacteria can function in the gut long-term as live diagnostics of inflammation or be used as prebiotics or probiotics (71, 72). These examples highlight only a few of the many applications, and while focused on enhancing warfighter performance and resilience, they should also be considered a platform to discover novel therapeutics extended for use in the general public.
